# Safety and Effectiveness of De-escalated Radiation Dose in T1-3 Nasopharyngeal Carcinoma: A Propensity Matched Analysis

**DOI:** 10.7150/jca.33303

**Published:** 2019-08-28

**Authors:** Xin Wang, Youyou Wang, Shengpeng Jiang, Jinlin Zhao, Peiguo Wang, Ximei Zhang, Fengming Wang, Zhenzhen Yin, Ping Wang

**Affiliations:** Department of Radiation Oncology, Tianjin Medical University Cancer Institute and Hospital, National Clinical Research Center for Cancer, Key Laboratory of Cancer Prevention and Therapy, Tianjin, Tianjin's Clinical Research Center for Cancer, Tianjin 300060, China

**Keywords:** Nasopharyngeal carcinoma, IMRT, Radiation dose, dose de-escalation

## Abstract

**Backgrounds**: With the excellent local control in T1 to T3 nasopharyngeal carcinoma (NPC) treated with intensity modulated radiotherapy (IMRT), the importance of toxicities is increasingly being recognised. This retrospective propensity score analysis sought to assess whether moderate dose reduction compromised long-term outcome compared with standard dose in T1-3 NPCs.

**Materials and Methods**: A total of 266 patients (67 female, 199 male) with a median age of 50 years between June 2011 and June 2015 were analysed. All were treated with IMRT, with or without systemic chemotherapy. The prescription radiation dose to gross tumor is 70Gy/2.12Gy/33F in our institution.

**Results**: With a median follow-up time of 50 months, the 5-year loco-regional failure-free survival (LRFS) and overall survival (OS) were 93.5% and 81.8%, respectively. 32 patients received radiation dose less than prescription dose, with a median dose of 63.6Gy (53-67Gy). Another 234 patients received exactly the prescription dose of 70Gy. Propensity scores were computed (32 patients treated with de-escalated dose and 64 patients with standard dose), there was no significant difference in 5-year LRFS and 5-year OS between the two groups (92.5% and 91.7% with standard dose; 82.1% and 85.7% with de-escalation dose; p=0.863 for LRFS and 0.869 for OS). No independent prognostic factor was associated with loco-regional failure in univariate analysis.

**Conclusions**: T1-3 nasopharyngeal carcinoma presenting with superior locoregional control, a moderately reduced dose (about 10%) delivered with IMRT resulted in comparable prognosis to those with prescription dose of 70Gy.

## Introduction

Nasopharyngeal carcinoma (NPC) is an endemic malignancy in China, showing highly radio- and chemo-sensitive 1. About 90% - 94% present with non-distant metastatic disease at their initial diagnosis 2, 3. Radiotherapy, as a definitive treatment modality, plays a crucial role in managing NPCs. In the era of two-dimensional radiotherapy (2D-RT), the 5-year overall survival (OS) was 59-69% and local recurrence-free survival (LRFS) was 60.8-79% 4. Intensity-modulated radiation therapy (IMRT) has been a major breakthrough in radiation techniques in recent decades, delivering a higher and more conformal dose to tumour volume that translates into better local control. IMRT is widely used in NPCs and several studies have reported 5-year LRFS at 86-92% and 5-year OS at 77-85% in large cohorts 5-7. Therefore, IMRT is recommended as the standard treatment of NPC, and about 10% gain in loco-regional controlling rates when compared with 2DRT.

In the setting of intensity-modulated radiotherapy, T-classification is no longer a significant prognostic factor of LRFS. No significant differences exist in loco-regional control in T1 to T3 disease, presenting with 5-year LRFS of around 90% in most of retrospective analyses 2, 3, 8. Radiation doses, to our best knowledge, have marked correlations to efficacy and toxicity. Radiation dose of >66Gy is normally regarded as tumoricidal and in adult NPCs, radiation dosages of around 70Gy to the primary tumor and 50Gy to the neck are considered the standard treatment 9. Combining the satisfying locoregional control in T1-3 NCPs and long-term RT-related toxicities lead us to question that dose 70Gy is still the standard dose in T1-3 NPCs treated with IMRT. Whether a moderate dose reduction would compromise long-term outcome compared with the standard dose? No clinical trial has been conducted to explore the feasibility of a relatively lower dose delivered by IMRT.

In our institute, the prescribed doses to primary tumor and metastatic lymph nodes in the treatment of NPCs are 70Gy, the same fraction as RTOG 0225^10^. In clinical practice, most patients completed the whole treatment as planned, only a small proportion did not due to poor tolerance. To assess whether reduced-dose radiotherapy would compromise the long-term loco-regional outcome, a study using propensity score analysis of T1-3 NPC treated with IMRT in our institute was conducted.

## Materials and Methods

### Patient selection

Between 2011 and 2015, the medical records of all NPC patients staged in T1-3NanyM0 treated with IMRT in our hospital were reviewed. The eligibility criteria were as follows: (1) histopathologically confirmed NPC; (2) radiologically measurable disease; (3) Karnofsky performance score (KPS) > 60; (4) complete baseline laboratory data with normal renal and liver function; (5) absence of pregnancy and lactation; (6) no distant metastases or concurrent malignancy; (7) no previous history of head and neck cancer; (8) no previous radiation to the head or neck. They were all re-staged using the 7^th^ edition of American Joint Committee on Cancer (AJCC) staging system, based on fibre optic nasopharyngeal scope observation, Magnetic Resonance Imaging (MRI) of the nasopharynx and neck, X-radiography or computed tomography (CT) of the chest, neck and abdominal ultrasonography, and emission computerized tomography (ECT) of bones. The study was approved by the Tianjin Medical University Cancer Institution and Hospital Reviewing Board. And a waiver for individual patients' consent for this retrospective study was also obtained from this committee. To maintain confidentiality, relevant medical records, laboratory results, images, and histopathological data were collected anonymously. The records of patients were kept confidential, and individuals outside this research team had no access to them.

### Radiotherapy

Simulated CT with axial images at 3mm intervals from the cranial apex to diaphragm was performed for each patient. A contrast enhancement scan was preferred to allow better visualisation of cervical vessels, except for patients with severe renal dysfunction, cardiovascular disease or allergic history to iodine. The image datasets were transferred to the PINNACLE planning system, version 9.8 (Philips Radiation Oncology Systems, Fitchburg, WI, USA).

Delineation and constraints were defined according to the consensus recommendations. Enhanced MRI of the nasopharynx was used for target contours. Gross target volume of the primary tumour (GTVp) and metastatic lymph nodes (GTVn) were defined as the visible tumor and involved nodes based on clinical, endoscopic and radiological examination. Clinical target volume 1 (CTV1) was defined as higher risk region, and covered nasopharynx, high-risk local structures (i.e., skull base, clivus, parapharyngeal space, retropharyngeal lymph nodes, sphenoid sinus, sphenomaxillary fossa, posterior part of the nasal cavity and maxillary sinus, and oropharynx), and positive lymph nodes and nodes at level IB (when nodes at level Ib were involved, and/or metastatic IIA LN size was more than 2cm), levels II, III, Va and VII. Clinical target volume 2 (CTV2) was defined as lower risk region, and included lymph nodes at levels IV, Vb and Vc as a prophylactic irradiated volume. The PGTVp was obtained by expanding the corresponding GTVp with a margin of 5 mm while limited by the brainstem, spinal cord, optic chiasma and optic nerve. The PGTVn was the GTVn with an expansion of 5 mm. Planning target volume 1 (PTV1) and 2 (PTV2) were expanded with a 3 mm margin of CTV1 and CTV2. IMRT was given in 33 fractions by use of a simultaneous integrated boost technique. PGTVp and PGTVn were delivered at 69.96Gy (2.12Gy per fraction) and PTV1 at 60.06Gy (1.82Gy per fraction) in 33 fractions, and PTV2 at 50.96Gy (1.82Gy per fraction) in 28 fractions. Radiation therapy was given on a conventional schedule of 5 daily fractions per week from Monday through Friday for 33 days. The details of the normal tissue constrains followed the protocol of Radiation Therapy Oncology Group (RTOG) trial 0225.

### Chemotherapy

Chemotherapy was part of the treatment for stage II to IVb patients provided that there were no contraindicating major medical co-morbidities. Various sequences and regimens (mostly cisplatin-based) were also used. The concurrent chemotherapy regimen consisted of intravenous cisplatin 75-100 mg/m^2^ delivered in 3 daily doses and administered every 21 days for each cycle. Chemotherapy doses and cycles were slightly adjusted according to adverse reactions. In addition to CCRT, individualised induction chemotherapy (IC) and adjuvant chemotherapy (AC) were used according to the characteristics of patients, disease stage and tolerance for the treatment with the principle of no more than 6 cycles of total chemotherapy. The chemotherapy mainly included three regimens: 1) docetaxel 75 mg/m^2^, d1, cisplatin 75 mg/m^2^, d1-3, and 5-fluoruouracil 750 mg/m^2^, d1-5, every 3 weeks; 2) cisplatin 100 mg/m^2^ and 5-fluoruouracil 1,000 mg/m^2^, d1-5 every 3 weeks; and 3) docetaxel 75 mg/m^2^, d1 and cisplatin 75 mg/m^2^, d1-3, every 3 weeks.

### Statistical analysis

All endpoints, including locoregional failure-free survival (LRFS), distant metastasis-free survival (DMFS), disease free survival (DFS) and overall survival (OS), were defined from the start date of the radiotherapy to the final follow-up date. Survival rates were estimated using the Kaplan-Meier method, and were compared using the log-rank test. P-value of less than 0.05 was considered statistically significant. Statistical analyses were performed using SPSS software package version 20.0. The Propensity Score Matching (PSM) method was used to control the balance between the dose de-escalation group and standard group. Matching covariates in the score scale included age, gender, T stage, N stage and chemotherapy. PSM was conducted by STATA 12.0.

## Results

### Patients' characteristics and treatment data

A total of 266 patients with T1-3NanyM0 were included in this analysis. The median age was 50 years (range 18-75 years), with a male predominance (76%, 199/266). The clinical stage distribution was T1 in 61 cases, T2 in 110 cases, T3 in 95 cases, N0 in 31 cases, N1 in 40 cases and N2-3 in 195 cases. 218 (82.0%) of these patients underwent chemotherapy and the median number of chemotherapy cycles was 4. 163 (61.3%) patients underwent induction chemotherapy (IC) and 181 (68.0%) underwent concurrent chemotherapy (CC). Patients' characteristics and treatment data are shown in Table 1.

### Treatment outcomes and patterns of failures

**Figure 1:**
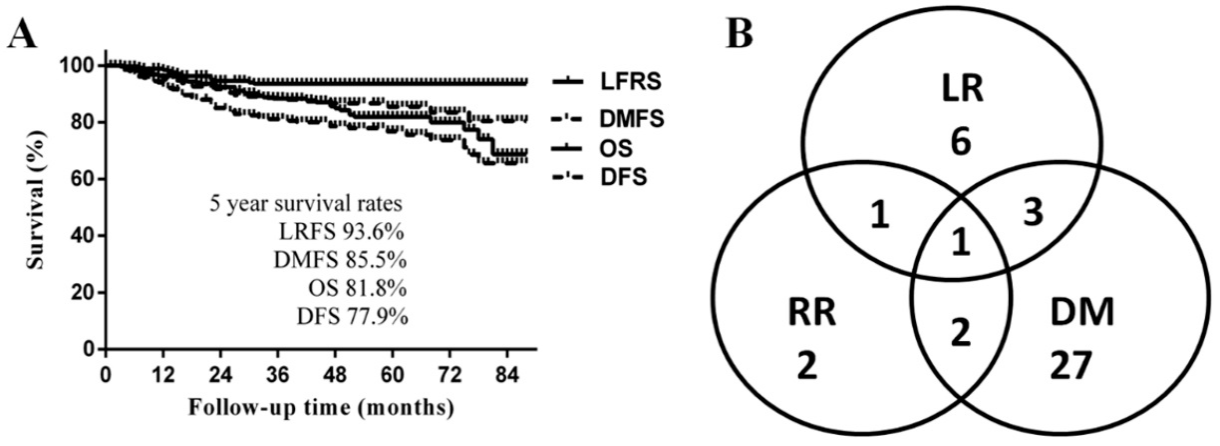
A. Survival curves of the whole cohort of patients; B Failure pattern of the whole cohort of patients

The median follow-up time was 50 months (range 3-89 months), and the 5-year LRFS, DMFS, DFS, and OS were 93.6%, 85.5%, 77.9% and 81.8%, respectively (Fig. 1A). A total of 42 patients (16%) developed disease progression. The most common failure pattern was distant metastasis, which occurred in 33 patients with a fairly high rate of 79%, and 9 (21%) developed locoregional recurrences only; the failure pattern is shown in Fig. 1B. By the last follow-up, 41 patients had died; 32 (78%) of disease progressions, mainly due to multiple distant metastases, and the other 9 patients of other reasons: 1 died a therapeutic-related death; 3 died of nasopharyngeal haemorrhage after treatment; 1 died of pneumonia; 1 died of a myocardial infarction; and the causes in 2 cases were unknown.

### Radiation dose and survival

Of the 266 patients, 32 did not complete the prescribed dose due to poor tolerance of severe acute toxicities, mainly severe mucositis. These 32 patients treated under 70Gy were included in the dose de-escalation group. The other 234 patients who received exactly 70Gy were categorized as the standard dose group. The median radiation dose in the de-escalation dose group was 63.6Gy (range, 53-67.8Gy), 17 (53.1%) were men and 15 (46.9%) were women. The median age was 49 years (range 22-73 years). The clinical stage distribution was T1 in 10 cases, T2 in 11, T3 in 11, N0 in 7 cases, N1 in 1 case and N2-3 in 24 cases. Some 25 (78.1%) of those patients underwent chemotherapy, and 20 (62.5%) underwent induction chemotherapy.

The proportion of women in the de-escalated dose group was higher (47% in the de-escalated dose group vs. 22% in the standard dose group, p=0.003), and there were more N0 cases (22% in the de-escalated dose group vs. 11% in the standard dose group, p=0.031). There were no significantly differences in other features. Patients' clinical characteristics and treatment data are shown in Table 2. The 5-year LRFS, DMFS, and OS in the de-escalation dose group and standard dose group were 92.5%, 89.5%, 82.1% and 93.7%, 85.0%, 81.7%, respectively, without significant difference (Table 3).

**Figure 2:**
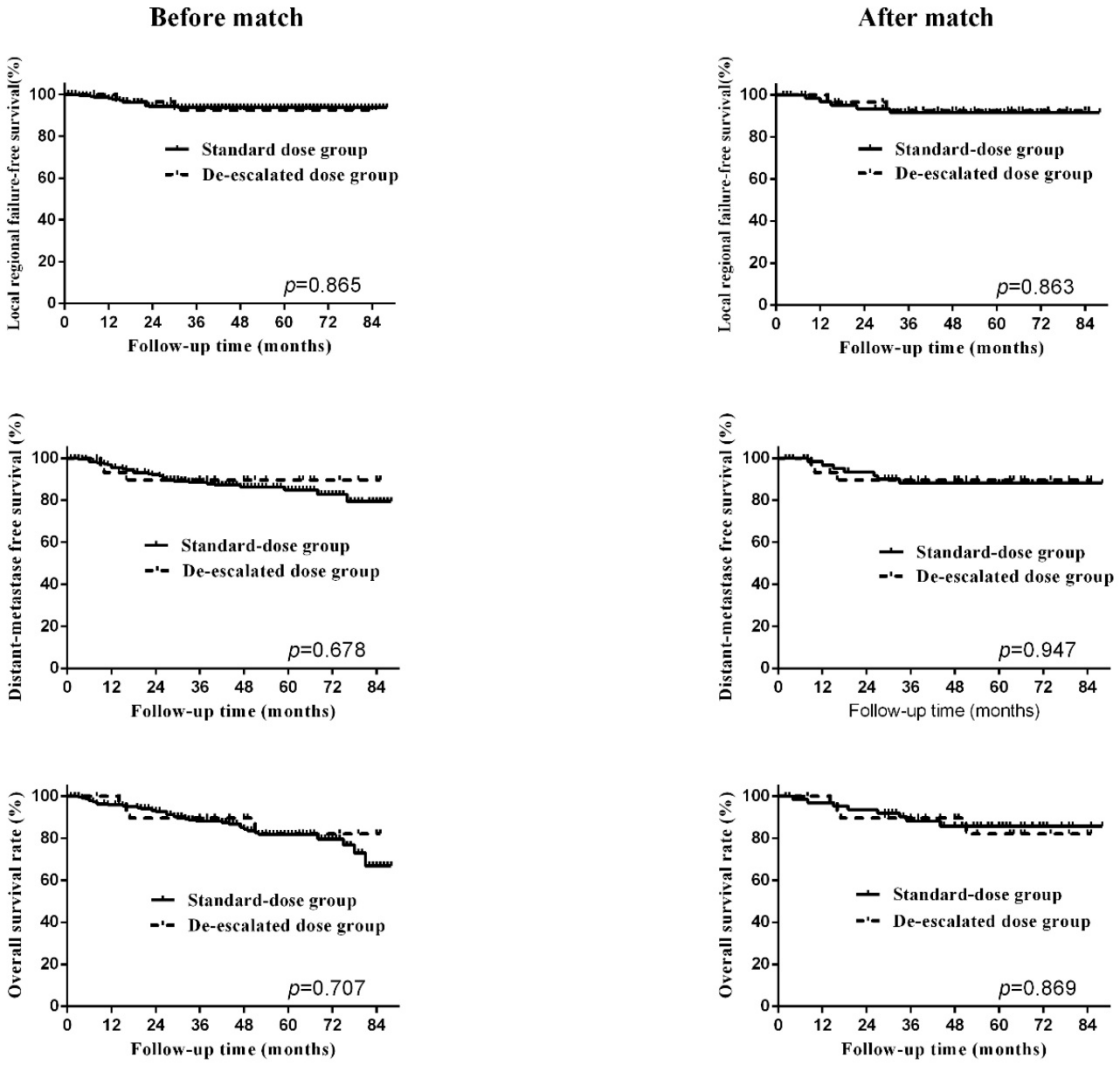
Survival curves, including locoregional-failure free survival, distant-metastasis free survival, overall survival, before and after matching.

To balance the biases between the two groups, propensity-matched analysis was conducted. After matching, the clinical characteristics and treatment data between the two groups were distributed evenly, shown in Table 2. The 5-year LRFS, DMFS and OS of the standard dose group were 91.7%, 88.1% and 85.7%, shown in Table 3, and no significant difference was observed between the two RT dose groups after matching. The survival curves before and after matching between two groups were shown in Figure 2. To further validate our findings, we again carried out PSM on these cases and reached similar results (Table S3 and Table S4).

### Prognostic factors

Univariate and multivariate analysis was conducted to determine the independent factors associated with LRFS and other endpoints. Unfortunately, no factor, including radiation dose, was associated with LRFS in the univariate analysis (Table 4), and thus, no multivariate analysis of LRFS was done. As to DMFS, DFS and OS, univariate and multivariate analysis results show that N stage was the only important independent prognosis factor, and there was no statistically significant difference between different radiation doses. The results of univariate and multivariate analyses are shown in Table S1 and Table S2.

## Discussion

IMRT was a great breakthrough in radiotherapy due to its dose modulating ability and steep dose gradient, improving dose conformity to the target volumes and minimising dose to the neighboring OARs (Organs at risks). In the era of IMRT, an excellent local controlling rate about 90% has been achieved in T1 to T3 disease 2, 3, 8. In our series, the 5-year loco-regional failure-free survival rates were 93.6% in T1-3 NPCs, which is consistent with other reports. No independent prognostic factors were found to be associated with LFRS. Such satisfying loco-regional controlling caused us to question whether the prescribed dose of 70Gy delivered with IMRT in adults was still necessary. Therefore, we collected patients who did not complete the prescribed dose due to poor tolerance. Surprisingly, no differences were found between patients treated with 63.6Gy (range, 53 -67Gy), and 70Gy before and after matching. It seems that radiation dose with about 10% reduction has no influence on long-term outcomes in T1-3 nasopharyngeal carcinomas.

Definitive radiotherapy is always the mainstay treatment in nasopharyngeal carcinoma. High dose radiation has a marked correlation with severe acute and long-term toxicities. Since long term complications such as xerostomia, endocrine defects, tissue fibrosis and secondary neoplasms would significantly affect quality of life (QoL) 11, 12, approaches to advanced treatment of NPCs should include strategies to further decrease treatment intensity, especially in T1-3 disease presenting with superior loco-regional controlling, where clinicians should try to decrease adverse effect and maintain the long-term survivals. A similar situation exists in low-risk HPV oropharyngeal carcinoma, presenting with a 5-year survival of 85-90% 13, 14. Recently, and various clinical trials in low-risk HPV+ oropharyngeal carcinoma have already focused on de-intensification treatment to improve QoL without compromising survival 14-16.

Compared with 2D-RT, IMRT itself is a valid technique to decrease toxicity due to its dosage advantage. The loco-regional controlling of NPCs is further improved, and morbidities including xerostomia and temporal lobe necrosis are significantly decreased 17-19. However, it is still far from enough. The next step is to lower incidence and severity of complications as much as possible. Three approaches exist to decrease the toxicity of high-dose radiotherapy in NPC: reducing treatment volume after the addition of induction chemotherapy (IC); re-assessing the role of concurrent chemotherapy with IMRT; and decreasing radiation dose in selected patients.

The first, IMRT with reduced volume after induction chemotherapy, results in dose-reduction in adjacent OARS. A randomised study conducted by Hongru Yang et al, suggested that IC could shrink tumour volume, and that after adjusting the GTV to the post-IC tumor, the dose to adjacent normal tissue will be significantly decreased 20. These will ultimately be translated to improved QoL, and reduced tumour volume seen in post-IC images seemed to have no influence on loco-regional failure. Reducing treatment volume after IC is an effective approach to decreasing radiotherapy-related toxicities to adjacent organs and maintaining locoregional control.

On the basis of findings from several randomised trials and meta-analyses, cisplatin-based concurrent chemo-radiotherapy has been considered the backbone in the treatment of loco-regional advanced NPCs 21, 22. However, due to the improvement in survival from IMRT, several studies in large cohorts have shown that there is no significant difference in prognosis in NPC treated with or without concurrent chemotherapy 3, 23. The benefits gained from concurrent chemotherapy were based on 2D-CRT and, mainly because of the improvement of locoregional controlling, not distant control. However, this therapeutic benefit did not continue in NPCs treated by adding chemotherapy to IMRT in most studies. Moreover, concurrent cisplatin presented with poor treatment compliance and decreased quality of life. Therefore, an increasing number of clinical trials have been conducted either omitting concurrent cisplatin (NCT 01817023, NCT 03015727, NCT 01854203), or substituting of other regimens of lower toxicity such as nimotuzumab (NCT02012062), carboplatin or nedaplatin^24^, ^25^. Omission or substitution will be one important method of reducing toxicity without compromising oncologic safety.

The last approach is also the most promising one: reduce radiation dose in selective NPCs with low risk of loco-regional recurrence. Radiation dose has been shown to be closely connected with long-term complications. Since long term toxicities are much tougher problems in child and adolescent patients than in adults, most radiation dose reduction clinical trials have conducted in young patients. Until now, 5 clinical trials have been demonstrated a 5-year EFS range from 77% to 91% in children and adolescent NPCs treated with induction chemotherapy followed by reduced-dose radiation, with the primary tumor site were between 54 and 68Gy and to the neck between 45 and 54Gy^26^-^30^. The GPOH-NPC study recommended that for patients with complete remission after IC, the radiation dose to the primary tumour can be safely reduced from 59.4Gy to 54.4Gy in children and adolescent NPCs 9. In adult NPCs, only one study has focused on radiation dose reduction in adults and found that 46 patients treated with 50Gy delivered by 2D-radiotherapy demonstrated a 5-year OS and LRFS of 74% and 73%, individually 31. Due to the small sample size in this study, the survival rate may not be representative, but it had confirmed that some radiosensitive NPC patients can be treated with 50Gy radiation. Our results, to our knowledge, is the first to demonstrate no decrease in locoregional control and survival in T1-3 NPCs with lower RT doses from 53 to 67Gy delivered by IMRT.

However, in a recent study by Ng et al. showed that GTV-P dose lower than 66.5Gy causes a significantly higher local regional failure 32. The 5-year LFFS was 90.4% and 54.3% for GTV_P66.5 <3.4cm^3^ and GTV_P66.5≥3.4 cm^3^. It concluded that local control would be significantly compromised when GTV_P66.5 exceeded 3.4cm^3^, which suggests that radiation dose is positively related to local control. The LFFS in T1, T2 and T3 were 100%, 89% and 87%, individually, significantly higher than T4 disease of 74%. However, the prescribed dose of 70Gy was delivered to at least of 95% of the GTV for the great majority of T1-3 patients, and most LR developed in T4 disease. Thus, the dosimetric inadequacy of the GTV_P66.5 may increase LR and remains a major problem with T4 disease, and it may not be applicable for a great majority of T1-3 disease. In our cohort, only T1-3 disease was selected for analysis, which presented only 6.4% of LRR at 5 years. Our results demonstrate that 10% dose reduction delivered with IMRT did not compromise long-term loco-regional control before and after matching. It is may be feasible if treated with 63.6Gy delivered by IMRT in T1-3 NPCs.

The main strength in this study was the use of PSM and multivariate analysis to evaluate the influence of radiation dose on the prognosis of T1-3 NPCs. It addressed the potential limitations of divergent confounders, treatment heterogeneity and selection bias associated with retrospective analysis of observational data. In terms of limitations, the data was derived from a single center and the sample size may be relatively small to address this issue. However, it is still the first important attempt to decrease toxicity by reducing radiation dose without compromising long-term survival in NPC treated with IMRT. In the future, we are looking forward to the findings in our study being validated in a larger sample of patients with a prospective multi-institution study.

## Conclusions

In our study of T1-3 nasopharyngeal carcinoma presenting with satisfying locoregional controlling, a moderate reduced dose (about 10%) delivered with IMRT resulted in comparable prognosis to those with prescription dose of 70Gy. The reduction in dose is meaningful because long-term toxicity is predominately correlated to total RT dose and a favorable long-term toxicity profile is expected with this de-intensified regimen.

## Supplementary Material

Supplementary tables.

## Figures and Tables

**Table 1:** Patients' characteristics and treatment data

Variables		Cases	Rates (%)	Variables		Cases	Rates (%)
Age	≤50	141	53	Gender	Male	199	75
	>50	125	47		Female	67	25
T stage	T1	61	23	N stage	N0	31	12
	T2	110	41		N1	40	15
	T3	95	36		N2-3	195	73
Clinical stage	I	6	2	RT dose	Standard dose	234	88
	II	45	17		De-escalated dose	32	12
	III	188	71	IC	Yes	163	61
	IV	27	10		No	103	39
CC	Yes	181	68	AC	Yes	162	61
	No	85	32		No	104	39

**Table 2:** Patients' characteristics and treatment data between standard dose group and de-escalated dose group before and after match

Variables			Before match		After match	
		De-escalated dose	Standard dose	p	Standard dose	p
Age	≤50	18 (56%)	123(53%)	0.695	38(59%)	0.770
	>50	14(44%)	111(47%)		26(41%)	
Gender	Male	17(53%)	182(78%)	0.003	43(67%)	0.264
	Female	15(47%)	52(22%)		21(33%)	
T stage	T1	10(32%)	51(22%)	0.467	17(27%)	0.888
	T2	11(34%)	99(42%)		24(37%)	
	T3	11(34%)	84(36%)		23(36%)	
N stage	N0	7(22%)	25(11%)	0.031	8(12%)	0.110
	N1	1(3%)	39(17%)		10(16%)	
	N2-3	24(75%)	170(72%)		46(72%)	
chemotherapy	Yes	25(78%)	193(83%)	0.548	54(84%)	0.450
	No	7(22%)	41(17%)		10(16%)	
IC	Yes	20(63%)	143(61%)	0.880	42(66%)	0.763
	No	12(37%)	91(39%)		22(34%)	

**Table 3:** Survival between standard dose group and de-escalated dose group before and after match

Survival		Before match		After match	
	De-escalated dose	Standard dose	p	Standard dose	p
LRFS	92.5%	93.7%	0.865	91.7%	0.863
DMFS	89.5%	85.0%	0.678	88.1%	0.947
OS	82.1%	81.7%	0.707	85.7%	0.869
DFS	75.9%	78.1%	0.881	82.1%	0.469

**Table 4:** Results of univariate analysis of LRFS.

Variables	HR	95%CI	p
Age	0.984	0.945-1.025	0.439
Gender	1.411	0.482-4.129	0.530
T stage			0.474
T2 vs T1	1.841	0.371-9.120	0.455
T3 vs T1	2.608	0.542-12.555	0.232
N stage			0.679
N1 vs N0	0.414	0.038-4.571	0.472
N2-3 vs N0	1.034	0.231-4.621	0.965
RT dose	0.974	0.818- 1.159	0.764
IC	1.338	0.485-3.691	0.574
CC	0.525	0.148-1.862	0.319
AC	0.405	0.114-1.436	0.162
