# Supplementary material for: Safety and Effectiveness of De-escalated Radiation Dose in T1-3 Nasopharyngeal Carcinoma: A Propensity Matched Analysis
Source: J Cancer. 2019 Aug 28;10(21):5057–64. doi: 10.7150/jca.33303 (PMC6775602; doi:10.7150/jca.33303)
Supplement: Supplementary file 1 — Supplementary tables. [file jcav10p5057s1.pdf]

Table S1 Results of univariate analysis of DMFS, OS and DFS.

	DMFS		OS		DFS	
Variables	HR(95%CI)	p	HR	p	HR	P
Age	0.017(0.987-1.048)	0.266	1.018(0.991-1.046)	0.220	1.015(0.992-1.038)	0.202
Gender	0.386(0.136-1.099)	0.075	0.408(0.160-1.040)	0.060	0.690(0.357-1.333)	0.270
T stage		0.084		0.070		0.162
T2 vs T1	0.446(0.176-1.132)	0.089	0.965(0.383-2.234)	0.862	1.091(0.526-2.263)	0.816
T3 vs T1	1.156(0.518-2.579)	0.723	1.960(0.860-4.468)	0.109	1.757(0.856-3.577)	0.120
N stage		0.047		0.059		0.022
N1 vs N0	0.796(0.050-12.743)	0.872	2.336(0.243-22.482)	0.463	1.651(0.302-9.025)	0.563
N2-3 vs N0	5.403(0.737-39.585)	0.097	6.231(0.855-45.416)	0.071	4.495(1.094-18.465)	0.037
RT dose	0.778(0.238-2.551)	0.619	0.821(0.292-2.303)	0.707	1.062(0.482-2.343)	0.881
IC	0.655(0.314-1.365)	0.258	1.035(0.551-1.943)	0.915	0.871(0.508-1.493)	0.614
CC	0.738(0.330-1.647)	0.458	0.803(0.390-1.654)	0.551	0.863(0.474-1.568)	0.628
AC	0.431(0.187-0.995)	0.049	0.613(0.306-1.225)	0.166	0.523(0.286-0.957)	0.036

Table S2 Results of multivariate analysis of DMFS, OS and DFS.

Variables	HR	95%CI	p
DMFS			
N stage	3.297	1.191-9.130	0.022
OS			
T stage	1.499	0.978-2.296	0.063
N stage	2.521	1.163-5.464	0.019
DFS			
N stage	2.316	1.253-4.277	0.007

Table S3 Patients' characteristics and treatment data between standard dose group and de-escalated dose group before and after match (1:3 match).

Variables			Before match		After match	
		De-escalated dose	Standard dose	p	Standard dose	p
Age	≤50	18 (56%)	123(53%)	0.695	46(58%)	0.904
	＞50	14(44%)	111(47%)		34(43%)	
Gender	Male	17(53%)	182(78%)	0.003	55(69%)	0.119
	Female	15(47%)	52(22%)		25(31%)	
T stage	T1	10(32%)	51(22%)	0.467	26(33%)	0.992
	T2	11(34%)	99(42%)		27(34%)	
	T3	11(34%)	84(36%)		27(34%)	
N stage	N0	7(22%)	25(11%)	0.031	17(21%)	0.874
	N1	1(3%)	39(17%)		5(6%)	
	N2-3	24(75%)	170(72%)		58(73%)	
chemotherapy	Yes	25(78%)	193(83%)	0.548	63(79%)	0.942
	No	7(22%)	41(17%)		17(21%)	
IC	Yes	20(63%)	143(61%)	0.880	53(66%)	0.707
	No	12(37%)	91(39%)		27(34%)	

Table S4 survival between standard dose group and de-escalated dose group before and after match (1:3 match).

Survival		Before match		After match	
	De-escalated dose	Standard dose	p	Standard dose	p
LRFS	92.5%	93.7%	0.865	88.7%	0.564
DMFS	89.5%	85.0%	0.678	82.5%	0.690
OS	82.1%	81.7%	0.707	86.8%	0.809
DFS	75.9%	78.1%	0.881	73.7%	0.931
